# Automated image transcription for perinatal blood pressure monitoring using mobile health technology

**DOI:** 10.1371/journal.pdig.0000588

**Published:** 2024-10-02

**Authors:** Nasim Katebi, Whitney Bremer, Tony Nguyen, Daniel Phan, Jamila Jeff, Kirkland Armstrong, Paula Phabian-Millbrook, Marissa Platner, Kimberly Carroll, Banafsheh Shoai, Peter Rohloff, Sheree L. Boulet, Cheryl G. Franklin, Gari D. Clifford

**Affiliations:** 1 Department of Biomedical Informatics, Emory University, Atlanta, Georgia, United States of America; 2 Center for Indigeous Health Research, Wuqu’ Kawoq — Maya Health Alliance, Tecpán, Chimaltenango, Guatemala; 3 Department of Gynecology and Obstetrics, Emory University, Atlanta, Georgia, United States of America; 4 Department of Obstetrics and Gynecology, Morehouse School of Medicine, Atlanta, Georgia, United States of America; 5 Division of Global Health Equity, Brigham and Women’s Hospital, Boston, Massachusetts, United States of America; 6 Department of Biomedical Engineering, Georgia Institute of Technology, Atlanta, Georgia, United States of America; McGill University, CANADA

## Abstract

This paper introduces a novel approach to address the challenges associated with transferring blood pressure (BP) data obtained from oscillometric devices used in self-measured BP monitoring systems to integrate this data into medical health records or a proxy database accessible by clinicians, particularly in low literacy populations. To this end, we developed an automated image transcription technique to effectively transcribe readings from BP devices, ultimately enhancing the accessibility and usability of BP data for monitoring and managing BP during pregnancy and the postpartum period, particularly in low-resource settings and low-literate populations. In the designed study, the photos of the BP devices were captured as part of perinatal mobile health (mHealth) monitoring programs, conducted in four studies across two countries. The Guatemala Set 1 and Guatemala Set 2 datasets include the data captured by a cohort of 49 lay midwives from 1697 and 584 pregnant women carrying singletons in the second and third trimesters in rural Guatemala during routine screening. Additionally, we designed an mHealth system in Georgia for postpartum women to monitor and report their BP at home with 23 and 49 African American participants contributing to the Georgia I3 and Georgia IMPROVE projects, respectively. We developed a deep learning-based model which operates in two steps: LCD localization using the You Only Look Once (YOLO) object detection model and digit recognition using a convolutional neural network-based model capable of recognizing multiple digits. We applied color correction and thresholding techniques to minimize the impact of reflection and artifacts. Three experiments were conducted based on the devices used for training the digit recognition model. Overall, our results demonstrate that the device-specific model with transfer learning and the device independent model outperformed the device-specific model without transfer learning. The mean absolute error (MAE) of image transcription on held-out test datasets using the device-independent digit recognition were 1.2 and 0.8 mmHg for systolic and diastolic BP in the Georgia IMPROVE and 0.9 and 0.5 mmHg in Guatemala Set 2 datasets. The MAE, far below the FDA recommendation of 5 mmHg, makes the proposed automatic image transcription model suitable for general use when used with appropriate low-error BP devices.

## Author summary

Monitoring blood pressure (BP) is critical during pregnancy and the postpartum period, especially in low-resource settings. Transferring BP data from devices to medical records poses significant challenges, particularly for low-literate populations. To address this, we developed an automated image transcription technique that accurately transcribes BP readings from photos of BP devices, making this data more accessible for healthcare providers. Our research involved capturing BP device photos as part of mobile health (mHealth) programs in rural Guatemala and Georgia, USA. Data were collected from pregnant and postpartum women, supported by local midwives in these regions. We designed a deep learning model that first locates the BP reading on the device screen using the YOLO object detection model and then recognizes the digits using a convolutional neural network. The model demonstrated high accuracy, with a mean absolute error significantly below the FDA’s recommended limit, proving its suitability for general use. This approach enhances the integration of BP data into health records, improving BP monitoring and management in low-resource and low-literate populations, ultimately contributing to better maternal health outcomes.

## Introduction

Hypertensive disorders of pregnancy (HDP) are the most common medical complication encountered during pregnancy [1]. HDPs are related to a combination of maternal, placental and fetal factors and can lead to serious complications which can cause maternal and fetal morbidity and mortality [2]. The burden of these complications is disproportionately borne by women in low and middle-income countries (LMICs) and resource-constrained areas of high-income countries. For example, in Latin America, pregnancy vascular disorders are the leading cause of maternal mortality where up to 26% of maternal deaths are estimated to be related to preeclampsia [3, 4]. In the USA, during 2017–2019, the prevalence of HDP among delivery hospitalizations increased from 13.3% to 15.9% [5]. This trend is particularly concerning given the existing disparities in maternal health outcomes across different regions. For example, Georgia has among the most disparate maternal health outcomes in the US with significant disparities in maternal morbidity and mortality rates and access to quality care [6]. These disparities are driven by a combination of social, economics and systematic factors [7–9]. Moreover, both the US and LMICs exhibit geographic and neighborhood-level disparities in hypertension burden [10]. These disparities highlight the importance of addressing systemic healthcare issues related to health equity in monitoring HDPs. Early detection, effective management, and timely referral to specialized care are essential to improve hypertension outcomes in pregnancy and reduce preventable maternal and fetal morbidity and mortality. However, there are limitations in management and control of hypertension in pregnancy which includes delay in the decision to seek care, failure to identify signs of high risk pregnancies along with a delay in responding to the clinical symptoms [11]. Traditionally, BP monitoring during pregnancy and postpartum is done through periodic visits to the healthcare provider. However, this approach may not always be feasible or practical, particularly in low-resource settings or for women with limited access to healthcare, and leaves gaps in care. The use of mobile health (mHealth) technology for BP monitoring during pregnancy and postpartum has the potential to address some of the challenges and disparities, enabling early detection and management of hypertension.

Routine BP monitoring has been shown to be an effective tool for identifying individuals at risk. BP self-measurement is often utilized as part of telemonitoring process that can help overcome issues related to poor healthcare access, white coat effect, and provide more detailed insights into the BP lability. However, this approach is also prone to errors through incorrect usage, poor choice of device and transcription and transmission errors [12]. In particular, most BP monitors have not been evaluated for operational accuracy in HDP, and those that have, often do not have easy and free Bluetooth connectivity [13]. This presents a key problem for home-based BP monitoring in pregnancy and elaborates the need for efficient and reliable methods for transcribing, reading, and transmitting data from standard BP devices.

**Fig 1:**
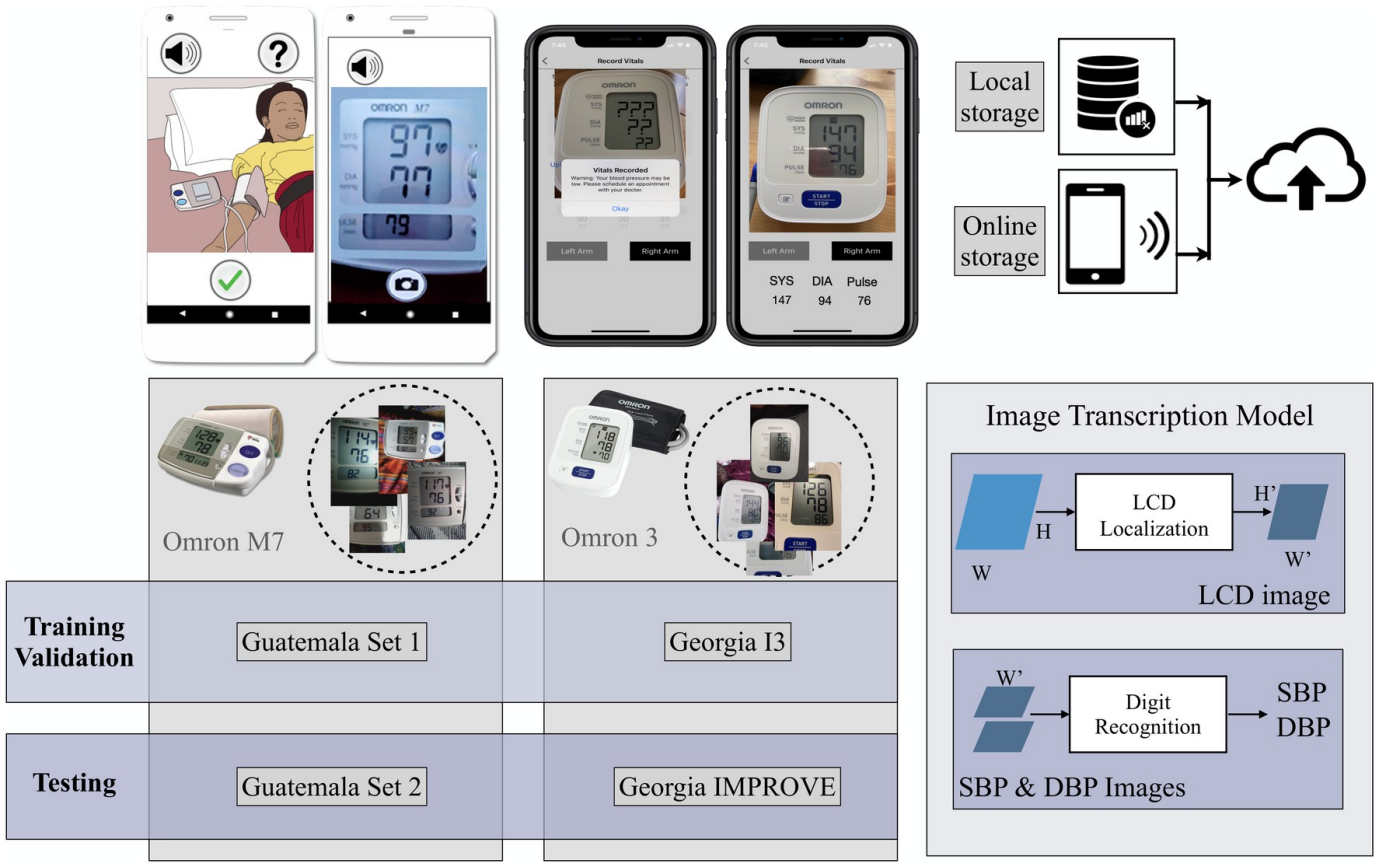
Overview of the data collection and the developed model for transcribing BP images. Upper left: The screenshots of the mobile apps employed in the Guatemala Perinatal, Georgia I3 and Georgia IMPROVE studies are shown. Upper right: In the Guatemala Perinatal study, images were initially stored locally before being uploaded to cloud storage. However, in the Georgia studies, data uploading was done directly from mobile devices. Lower left: Datasets utilized for model training, validation, and testing, including Guatemala Set 1, Guatemala Set 2, Georgia I3, and Georgia IMPROVE are identified. The studies employed an Omron M7 automated oscillometric BP monitor for the Guatemala datasets, and Omron 3 devices for the Georgia datasets. Lower right: The BP image transcription model consists of two steps: 1) LCD Localization and 2) Digit recognition.

In this study, our goal is to develop a low-cost and accessible mHealth system with automatic AI-based transcription of BP from LCDs to address the challenges of BP monitoring during pregnancy and postpartum in populations with low literacy levels, high rates of HDP and limited access to healthcare. To achieve this goal, we developed an automatic BP image transcription model and evaluated the model’s performance across multiple datasets and with the varying BP device types available to our health care workers, considering the FDA recommendation of less than a 5 mmHg error. Specifically, the developed BP image transcription model was trained and validated using the data collected in two countries, including in perinatal monitoring study in Guatemala [14–16] and postpartum BP monitoring studies in Georgia, USA. Fig 1 shows the overview of the developed model and datasets used in each phase of training/validation and testing the model.

In the designed mHealth system the transmission of the BP measurement is based on using ubiquitous cell phone cameras. We have developed a deep-learning-based digit recognition model that automates the transcription of the images (Fig 1). In earlier work, we showed that deep-learning-based digit recognition from photos of BP device displays can accurately capture such data [17]. Building upon our previous work, this study represents an enhancement of the model. Specifically, in this work we have used the YOLO (You Only Look Once) [18] object detection model to locate LCDs in the images to address the challenges observed in the contour-based model. The limitations of the contour-based LCD detection included reduced robustness when facing variations in angle, distance from the camera, lighting condition, and device types. The adoption of the YOLO model brings a notable advantage as it demonstrates an impressive ability to identify LCD displays in images of varying quality and across different device types. Another contribution of this study involves the extensive expansion and comprehensive evaluation of our automatic transcription model’s applicability across different device types, study designs, and diverse populations. This extensive exploration aims to identify and establish a more robust training strategy, further enhancing the system’s effectiveness and reliability.

The proposed model includes LCD localization, pre-processing and classification of digits and has the potential to be applied in a wide range of applications where digit recognition in LCDs is needed, such as in glucose or weight measurement devices. By monitoring BP outside of clinical visits, and outside of office hours, we have the potential to capture BP in pregnancy and postpartum at critical, typically unmonitored, times. This mHealth solution is entirely scalable with no required specialized equipment and has the potential to improve maternal and fetal outcomes by enhancing access to accurate BP monitoring in low resource settings.

## Background

### mHealth system for BP monitoring

mHealth BP monitoring systems have demonstrated superior performance in comparison to traditional methods of BP monitoring, particularly in terms of convenience and management of hypertension [19, 20]. In these monitoring systems, once BP data has been measured, several methods allow users to record and transmit this data to clinicians. Core elements of number digitization are manual transcription on both paper and smartphone, Bluetooth or cellular data receivers and memory-card based and USB transfer. Each of these approaches has potential benefits and drawbacks, particularly in terms of risk of missing and inaccurate data. In the manual transcription, users may introduce errors during the transfer of data from the device display [21]. Furthermore, even trained clinical experts make significant errors when transcribing medical information [22]. Transferring the data using wireless BP devices is also prone to connectivity errors due to interference, variations in standards and various installed apps and services interfering with the connection. Memory card-based storage and USB transfer also introduce complications due to using cables. More importantly, given the implications of inaccurate BP measurement, validation of BP devices in hypertensive populations, especially perinatal populations, should be verified. The definitive work evaluating devices in hypertensive populations identified only a very small number of devices which are appropriate for preeclampsia, and none with wireless connectivity [13].

Automatic transcription of the images can facilitate transferring the data from home-based BP monitoring system, especially in populations with lower educational attainment and who are less likely to use mHealth tools, potentially due to challenges related to digital literacy.

### Digit recognition

Digit recognition, involving the identification of handwritten or printed digits through the utilization of machine learning algorithms, finds practical application in mHealth systems by facilitating automatic transcription. Optical Character Recognition (OCR) is one of the essential computer vision applications which involves converting images to editable and searchable digital documents. OCR technology has been in use since the 1980s [23–25], and it continues to advance with the integration of state-of-the-art methods. Over the years, digit recognition algorithms have shown substantial improvements, using various classifiers such as support vector machines [26], k-nearest neighbors [27], and more recently, deep learning techniques. Among these, convolutional neural networks (CNNs) have demonstrated exceptional performance in digit recognition across diverse applications [28–30].

Furthermore, commercial solutions like Google Vision OCR have gained prominence in the field of OCR technology. However, they have generally been optimized for scanner-captured documents rather than camera-captured documents or images [31]. Image-based OCR tools include Tesseract OCR [32, 33], Abbyy Mobile OCR Engine, and mobile applications such as CamScanner and My Edison [34]. While some of these methods provide quick and affordable data digitization, their accuracy drops significantly for images with distortions, noise and unusual characters [17, 31]. In particular, there is virtually no research in the recognition of digital characters formed by seven disjoint elements, which are common to LCD devices. (See Fig 1, top left, for an example of such an image.) In our research, we have adopted an image-based OCR approach employing CNNs for accurate recognition of sequences of digits displayed on LCD screens.

## Data collection

**Fig 2:**
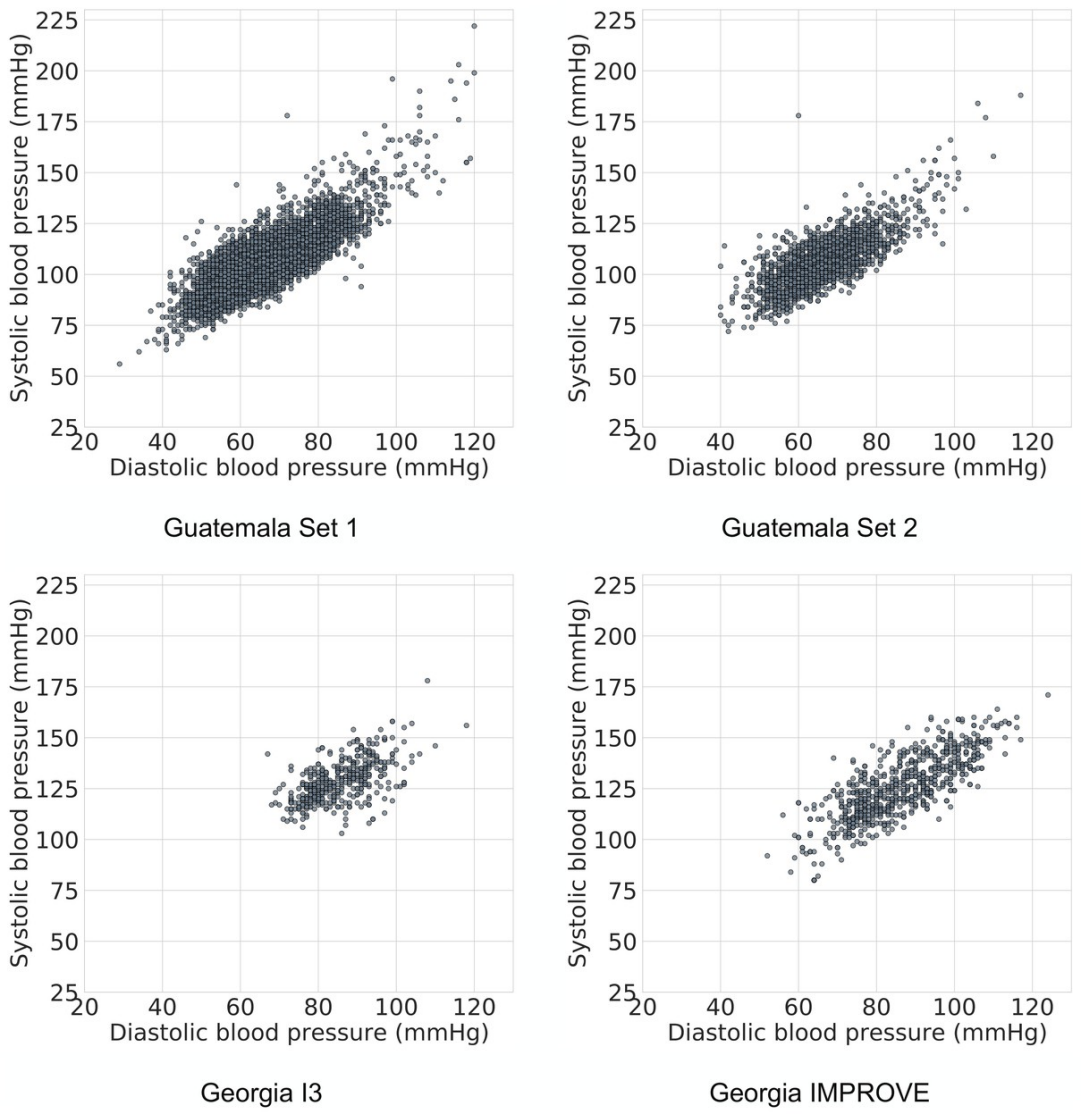
Distribution of BP data in the four datasets used in this study. The demographics of the individuals are given in Table 1.

In this study we trained and validated the BP image transcription model on four datasets which are described below. A summary of the datasets is provided in Table 1 and the distribution of BP readings is provided in Fig 2.

**Table 1:** Datasets used for model training, validation, and testing.

Datasets	Number of patient	Number of images	Location	Time of recording	Inclusion criteria
Guatemala Set 1	1697	8192 7205 readable	Highland Guatemala Tecpan, Chimaltenango	Second and third trimesters	Indigenous Maya
Guatemala Set 2	584	1934 1744 readable	Highland Guatemala Tecpan, Chimaltenango	Second and third trimesters	Indigenous Maya
Georgia I3	23	475 427 readable	Grady Hospital Georgia, US	Up to 6 weeks postpartum	African American 18 years or older with HPD
Georgia IMPROVE	49	776 720 readable	11 sites in Georgia, US	Up to 3 months postpartum	African American 18 years or older

### Guatemala Set 1

The Guatemala Perinatal mHealth Intervention study was conducted in rural areas of Guatemala to identify changes in outcomes of pregnant women due to the use of an Android mHealth app [14–16]. At each visit, a traditional birth attendant recorded at least two maternal BP recordings using the Omron M7 (Omron Co., Kyoto, Japan) self-inflating device and captured the photo of the BP device using the developed mobile application (Fig 1). Visits were conducted in a mother’s home, where there might be poor lighting conditions. The user was trained to align the image using a mask that appears in the app for capturing the photo. Between January 2013 and July 2019, a total of 8,192 images were captured from 1,697 pregnant women carrying singletons between 6 weeks and 40 weeks gestational age. Before processing the images, the systolic BP (SBP), diastolic BP (DBP), and heart rate (HR) of each BP image were manually transcribed by three independent annotators. Annotators screened each of the images for readability as well as image quality labels. Readability was defined as the ability to clearly transcribe the full numerical values. A total of 7,205 images were annotated for the values of SBP, DBP, HR along with a quality label. Segregation of these images based on their quality metric yielded 1,261 “Good Quality” images and 5944 poor quality images (inclusive of images with “Blur,” “Dark,” “Far,” “Contains Reflections,” and “Cropped” quality labels).

### Guatemala Set 2

**Fig 3:**
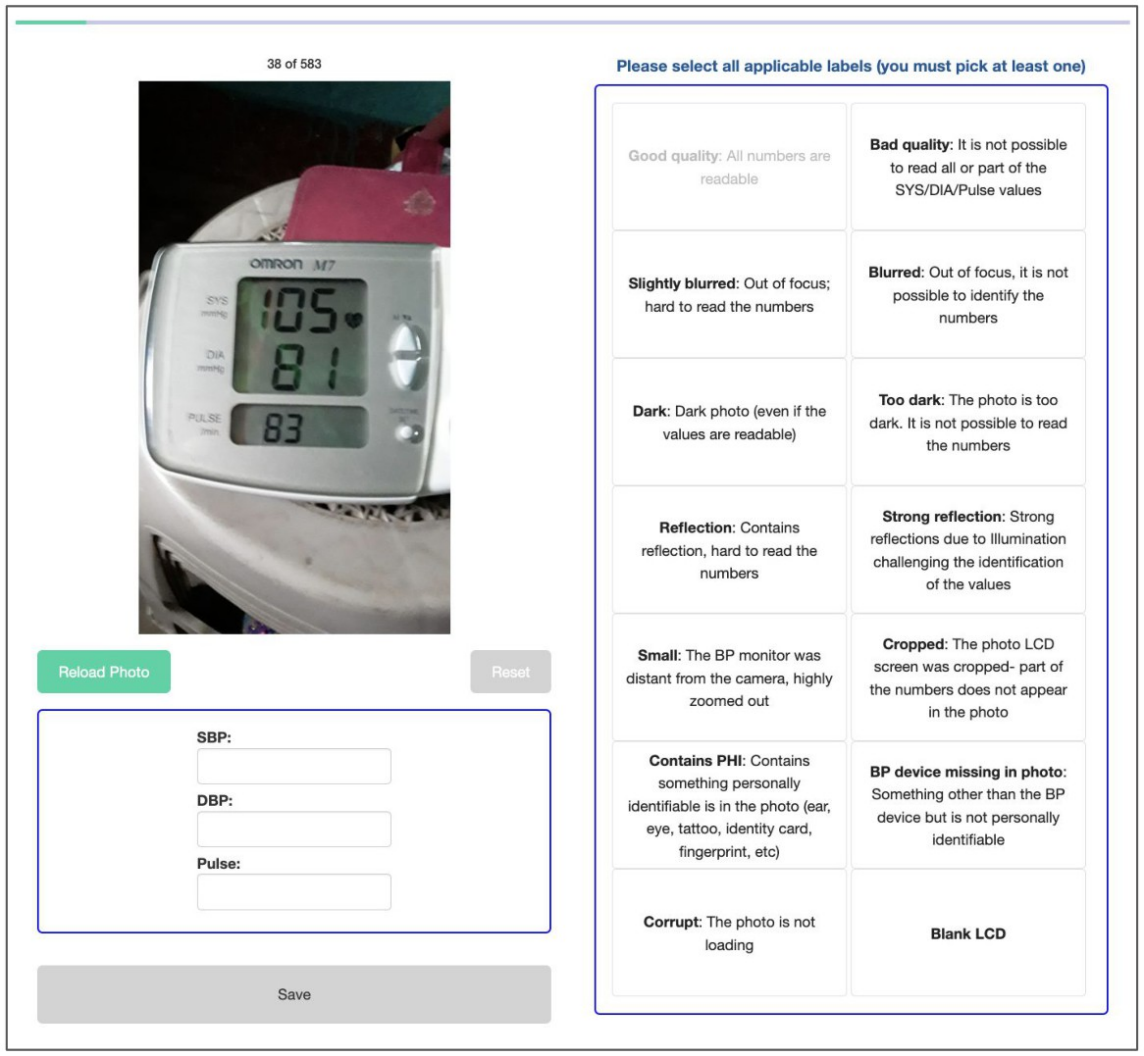
The designed labeling web interface for transcribing and labeling the quality of the images in the Guatemala Perinatal study.

Between August 2019 and October 2021, a total of 1934 blood pressure (BP) recordings were collected from 570 pregnant women by 28 midwives in Guatemala. The BP images were annotated by 10 independent annotators, with each image being labeled by three annotators. The labeling web interface was designed to collect the SBP, DBP and heart rate, with each of them being manually transcribed by the annotators. Additionally, the annotators labeled the quality of the images by choosing the defined quality labels. Fig 3 shows the examples of the app screens and the labeling interface.

### Georgia I3

**Fig 4:**
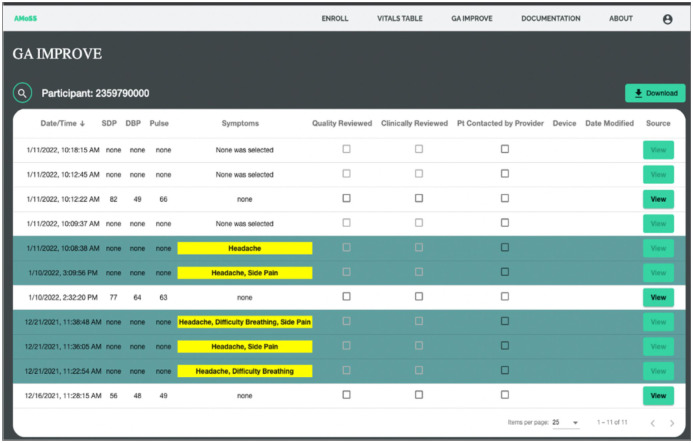
The backend dashboard used in the Georgia I3 and Georgia IMPROVE studies for postpartum BP monitoring.

The Georgia I3 was conducted in an urban setting in Georgia, US to study the feasibility of mHealth BP monitoring for the early detection of exacerbation of hypertension. Participants were postpartum women 18 years or older who delivered a liveborn infant at Grady Memorial HospitSet1al in Atlanta, Georgia and were diagnosed with hypertensive disorders during pregnancy or at delivery. Consenting participants were given an Omron BP710N Series 3 upper arm BP monitor. Participants measured their BP twice daily for a 6-week period. They used the smartphone application (Moyo Mom) created by our team to capture the photo of the BP device and transcribe the numbers manually. A clinician had access to the participant data via a backend interface that serves as a case management portal (Fig 4). A study coordinator reviewed the collected data labeled the images as “study device”, “not study device” and “unidentified”. A study coordinator validated/corrected the numbers entered by participants using the designed clinical dashboard. The data were collected between March 2021 and November 2021 and consists of 475 BP images recorded by 23 participants. 427 images were captured from study devices, 36 from other BP devices and 5 were from unidentified BP devices.

### Georgia IMPROVE

The Georgia IMPROVE study was designed to determine the association between cardiovascular complications during perinatal period, postpartum depression and symptoms of Covid-19. We recruited women 18 years or older during the late third trimester or early postpartum period in 11 sites in Georgia, including the Grady Memorial Hospital. The Moyo Mom app, used in the Georgia I3 study, was adapted for the IMPROVE study. The app, which is available on both iOS and Android platforms, is designed for participants to self-report metrics including but not limited to symptoms related to severe hypertension, effects of COVID-19 as well as personal experiences related to mental health, structural racism and discrimination. Participants are able to upload pictures of their BP readings which are then reviewed for accuracy by study coordinators and clinicians using the clinical dashboard. We have incorporated several alert features into the app design process in order to notify providers of potential poor outcomes, such as repeated high BP (SBP>160 mmHg or DBP>110 mmHg) readings, a reported symptom of severe hypertension and when a participant indicates self-harm ideation during the Mood Survey. Similar to Georgia I3, a study coordinator reviewed the collected data using the designed clinical dashboard. The data was collected between March 2022 and November 2022 and includes 776 images from 48 participants where 720 images were captured from the study device, 6 other BP devices and 49 were unidentified BP devices.

## Method

In this section the step-by-step approach to convert BP images into numerical format is described including LCD localization and the digit recognition methods.

### Automatic LCD localization

Accurately localizing the LCD frames is essential for converting the images into a numerical format, but this can be challenging due to orientation and zooming effects, resulting in differences in the size and location of the frames. Over the past decade, rapid advancements in deep learning have driven extensive research and significant contributions aimed at improving the performance of object detection. The YOLO model, currently represents the state of the art in this domain and have demonstrated success in accurately localizing objects in images in different applications such as detection of vehicles to improve transportation systems [35], surveillance and security [36], medical imaging [37], agriculture [38] and document processing [39].

In this work, to perform LCD localization using YOLO, the model was re-trained on a dataset of BP images for the task of LCD detection. During the training process, the model divides the image into a grid of cells and predicts the likelihood that an object, i.e., the LCD display, is present in each cell. The model also predicts the coordinates of the bounding box that surrounds the object, resulting in precise localization of the LCD display within the image.

### Digit recognition

**Fig 5:**
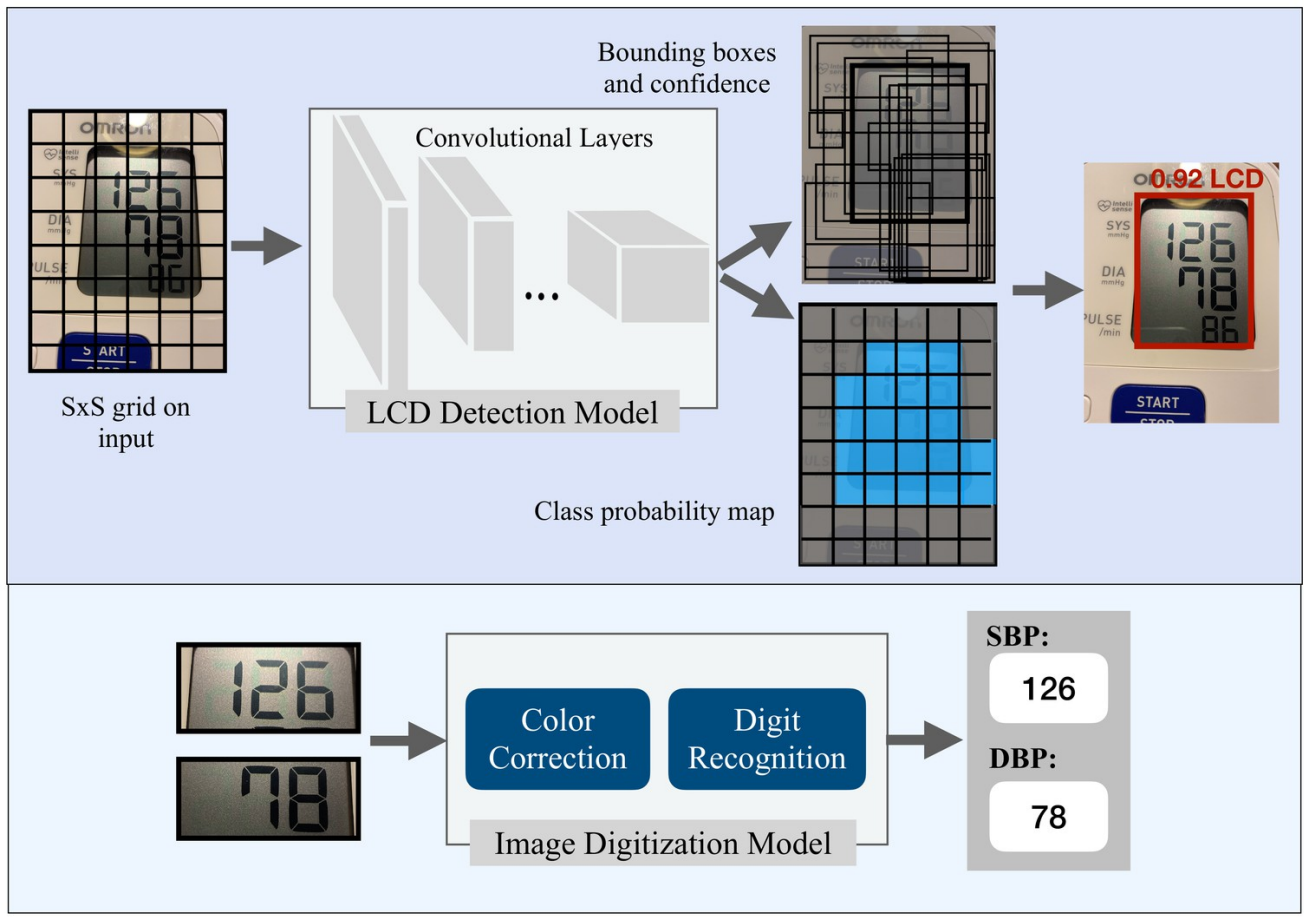
Overview of the BP image transcription. The LCD detection step uses the YOLO object detection model (top) and the CNN-based digit recognition model is for extracting numbers from SBP and DBP images (bottom).

Our approach to transcribe the BP images is based on the recognition of sequence of digits in the LCD images. Specifically, we aim to learn a model of *P*(*S*|*X*) where *S* represents the output sequence and *X* represents the input image. To model *S*, we define it as *N* random variables *s*_1_, *s*_2_, …, *s*_*N*_ representing the elements of the sequence. In the task of BP transcription the maximum value of BP is a 3-digit number, therefore *N* is chosen to be 3 and each digit variable has 10 possible values. An additional “blank” character was incorporated for shorter sequences. In the preprocessing step of the developed digit recognition model we applied a bilateral filter to smooth the images while preserving edges. Then, the images were fed to the gamma correction to reduce the effect of the illumination levels. In this work, the CNN-based model [17] was used to detect the sequences of digits. In this model a softmax classifier, receives extracted features from X by a CNN and returns the probability of each digit (Fig 5).

### Experimental setup

The bounding box of the LCDs were annotated in 80 images from Guatemala Set 1 and Georgia I3 datasets (40 images per dataset) using the LabelImg toolbox [40]. Subsequently, the YOLO V5 model was re-trained using the labeled dataset for the task of LCD detection. Using the extracted LCDs, the SBP and DBP images were created and resized to a matrix size of 180 x 80. In the digit recognition model, a three-layer CNN architecture with 32, 64, 128 filters of dimension 5x5 was used and each layer was followed by batch normalization, ReLU activation, and maxpooling. The resulting feature vector from the CNN was then fed into three softmax classifiers.

To optimize the model parameters, a sparse categorical cross entropy loss function and mini batch stochastic gradient descent were used. In our model training process, we employed a batch size of 50, an initial learning rate of 0.001, and a learning rate decay mechanism that reduced the learning rate by a factor of 10 during training. Additionally, we implemented early stopping with a patience of 10 epochs, which allowed us to monitor and halt training. The best model was saved based on validation loss, ensuring that we retained the most optimal configuration for subsequent evaluation and analysis. The contour-based LCD detection and digit recognition models has been made available through an open-source licensing, as detailed in [41].

### Model evaluation

In the model evaluation, first, we compared the performance of the BP transcription using two different LCD localization methods: YOLO-based and contour-based [17] LCD detection.

**Fig 6:**
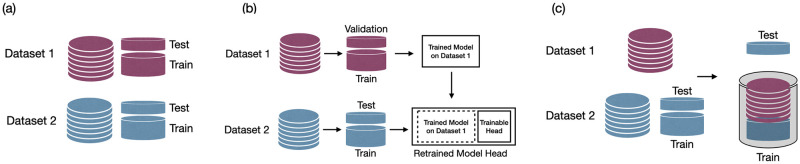
Figure depicting experimental setups for model performance evaluation: a) Device-Specific: Separate models trained and validated for each BP device using a cross-validation approach. b) Device-Specific with Transfer Learning: Utilizing a pre-trained model on Dataset 1 (dark red) during the cross-validation process, followed by fine-tuning on Dataset 2 (blue) for evaluation on Dataset 2. c) Device-Independent: Employing cross-validation with data concatenation from both datasets in the training subset.

Additionally, we investigated the effect of including LCD images extracted from two different BP devices (the Omron M7 and Omron 3) in the training phase. The performance of the digit recognition model was evaluated by defining three experiments. We conducted model evaluation through a five-fold cross-validation procedure applied to a randomly chosen subset of the images. As mentioned in the data collection section, the training and validation of the model were conducted using images captured from Omron M7 devices in the Guatemala Set 1 and Omron 3 BP devices in the Georgia I3 datasets. We conducted a series of experiments to evaluate the performance of our model, as illustrated in Fig 6. Following is the details of performed experiments:

- **Device-Specific**: Separate models were trained for each of the BP devices. Guatemala Set 1 dataset was used to train the digit recognition model for the Omron M7 device and the Georgia I3 dataset was used for the device-specific model corresponding to the Omron 3 BP device. The five fold cross validation was used to assess the performance of the model.
- **Device-Specific with transfer learning**: In this experiment, training the model was based on using transfer learning. Specifically, we used a pre-trained model and fine-tuned the model. For example, in the digit recognition from the Omron M7 BP device, we used the model trained on images of Omron 3 and re-trained the model.
- **Device-Independent**: In this experiment, we merged the LCD images from both datasets and trained a single model. To evaluate the performance of the device-Independent model, we conducted a five fold cross validation on each dataset. In this evaluation, we included the images from the other BP device in the training set to assess the model’s robustness.

To evaluate the transcription performance, we used two evaluation metrics: classification accuracy and mean absolute error (MAE). Classification accuracy was defined as the percentage of correctly transcribed BP values out of the total number of samples. We also calculated the MAE between the automatic transcriptions generated by the model and validated BP values provided by the study coordinator or annotators depending on the dataset. Once the final model was trained, we utilized it to transcribe the images in the test datasets. The overview of the datasets used for training, validation and testing the model is presented in Fig 1. To determine the statistical significance of the results, we performed the rank sum test to compare the manual and the automatic transcriptions. The null hypothesis was that there was no significant difference between the two methods, while the alternative hypothesis was that the automatic transcription was significantly different from the manual transcription.

## Results

**Fig 7:**
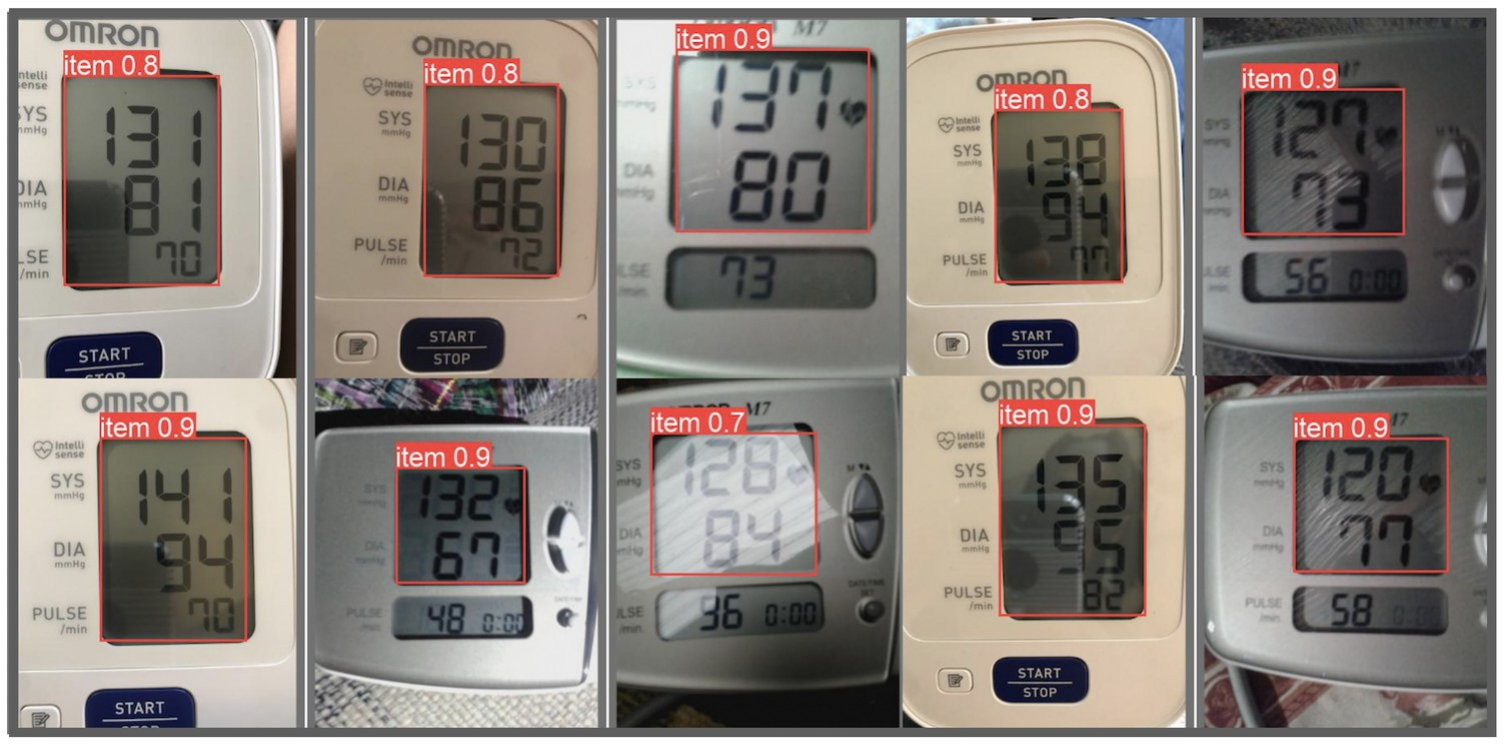
Examples of LCD detection results on images captured from Omron M7 and Omron 3 devices.

We compared two different LCD localization methods and their impact on BP transcription accuracy. The YOLO-based method and the contour-based method were tested on the same set of data previously used in a study by Kulkarni et al. [17]. Our results show that the YOLO-based method outperformed the contour-based LCD localization method as shown in Table 2. In this experiment, the model was trained on 5020 single LCD images and tested on 1677 images. The results of BP transcription, showed that the YOLO-based method improved both the accuracy and MAE of transcribing SBP and DBP. This suggests that the YOLO-based method is more accurate in detecting LCDs in the images which leads to having better performance in BP transcription reducing the MAE of SBP and DBP detection to 1.04 and 0.91 mmHg, respectively. Fig 7 illustrates examples of the bounding boxes around the LCD screens detected using the YOLO-based method, along with the corresponding confidence scores generated by the model.

**Table 2:** Comparison of contour-based LCD localization and YOLO object detection method in performance of the BP image transcription in Guatemala perinatal data.

LCD localization method:	Contour-based		YOLO-based	
Evaluation metrics	Acc	MAE	Acc	MAE
SBP	90.7	3.19	**93.7**	**1.04**
DBP	91.1	0.94	**96.6**	**0.91**

Our evaluation of the digit recognition model using three training strategies, as described in the “Model Evaluation” section, is summarized in Tables 3 and 4. The results obtained through five-fold cross validation on the Guatemala Set 1 and Georgia I3 (Table 3), indicates that the best-performing models were achieved using transfer learning for each BP device and device independent model trained on images from both devices. In the training and validation phase, both transfer learning and Device-Independent models have better performance than the Device-Specific approach. For the Guatemala Set 1 dataset, in the estimation of SBP, the Device-Independent approach achieved the lowest MAE at 1.4±1.5 mmHg. For DBP estimation, both Transfer Learning and Device-Independent models yielded comparable results, with MAEs of 0.8 (±0.1 and ±0.2 standard deviations, respectively). For the Georgia I3 dataset, transfer learning model outperformed other approaches, with the lowest MAEs of 0.5±0.4 and 0.4±0.4 mmHg for SBP and DBP estimation. We applied the rank-sum test to assess statistical significance. In the case of the Guatemala Set 1 dataset, we observed that there was no statistically significant difference between the MAEs of the Device-Specific and Transfer Learning models (*p* = 0.1), as well as between the Device-Specific and Device-Independent model (*p* = 0.08). On the other hand, when we conducted the rank-sum test on the results from the Georgia I3 dataset, we found a statistically significant difference between the Device-Specific and Device-Independent models (*p* = 0.04). No statistical difference was found for the Device-Specific and Transfer Learning approach.

**Table 3:** Five-fold cross validated results of the digit recognition model using three training strategies. Accuracy (Acc) is in percent and Mean Absolute Error (MAE) is in mmHg.

	Device-Specific		Device-Specific+Transfer Learning		Device-Independent	
Evaluation metrics	Acc	MAE	Acc	MAE	Acc	MAE
Guatemala Set 1-SBP	94.3±1.8	1.5±0.6	94.4±1.7	1.5±0.5	94.2±1.9	1.4±0.5
Guatemala Set 1-DBP	93.8±2.4	0.9±0.4	94.3±1.7	0.8±0.1	94.1±1.7	0.8±0.2
Georgia I3-SBP	94.1±5.5	0.6±0.6	96.9±2.9	0.5±0.4	96.2±3.2	0.7±0.8
Georgia I3-DBP	92±6	1.4±1.3	96.2±3.5	0.4±0.4	95.7±4.3	0.5±0.6

**Table 4:** Testing the top model across folds on held-out test datasets. Accuracy (Acc) is in percent and Mean Absolute Error (MAE) is in mmHg.

	Device-Specific		Device-Specific+Transfer Learning		Device-Independent		Average Human Transcription	
Evaluation metrics	Acc	MAE	Acc	MAE	Acc	MAE	Acc	MAE
Guatemala Set 2-SBP	95.1	1.0	96.2	0.8	96.6	0.9	93.1	4.1
Guatemala Set 2-DBP	96	0.6	95.6	0.7	96.7	0.5	92.6	2.7
GA IMPROVE-SBP	90	2.6	96.1	0.8	96.3	1.2	96.8	0.5
GA IMPROVE-DBP	89.1	2.1	92.7	2	96.3	0.8	96.9	0.3

In the next step, we assessed the performance of the optimized models on two held out test datasets, the Georgia IMPROVE and the Guatemala Set 2, as detailed in Table 4. We employed the MAE of SBP and DBP transcription as a metric to determine the top model across folds from the training/validation datasets. Overall, the Device-Specific model with Transfer Learning and Device-Independent models demonstrated the best performance. The result of the transfer learning approach was reported as MAE of 0.8 mmHg for SBP and 0.7 mmHg for DBP in the Guatemala Set 2 dataset and 0.8 mmHg, 2 mmHg for SBP and DBP in the Georgia IMPROVE dataset. And, using the device independent model the MAE was 0.9 mmHg for SBP and 0.5 mmHg for DBP in the Guatemala Set 2 dataset and 1.2 mmHg and 0.8 mmHg for SBP and DBP in the Georgia IMPROVE dataset. We applied the rank-sum test on the results of the test datasets (Guatemala Set 2 and Georgia IMPROVE) and we found a statistically significant difference between the Device-Independent and Transfer Learning methods (*p* = 0.009). However, the test did not show a statistical difference between the Device-Specific and Device-Independent models.

These results demonstrate the capability of the developed model to accurately transcribe BP images. Our analysis indicates the superiority of incorporating images from two types of BP device, whether through the transfer learning approach or by merging the datasets in the training phase. The model’s performance on both held out test datasets underscores its effectiveness in capturing and generalizing important features, enabling it to provide precise SBP and DBP predictions.

We compared the manual and automatic transcriptions in the Georgia IMPROVE and the Guatemala Set 2 datasets. The results of the Device-Independent model were used in this experiment. Detailed information regarding the manual transcription and validation of BP values for each dataset are provided in the “Data Collection” section. In the Georgia IMPROVE study, participants were instructed to input their BP values after capturing a photo of the device, and the study coordinator subsequently validated these transcriptions. For the Guatemala dataset, the data was transferred to our HIPAA compliant backend, where each image was labeled by three annotators. Table 4 presents the MAE and accuracy metrics for an average human transcription. In the IMPROVE study, the analysis of manual transcription of SBP and DBP values resulted in an accuracy of 96.8% and 96.9%, respectively. The corresponding MAE values were 0.5 mmHg for SBP and 0.3 mmHg for DBP. In the Guatemala study, the accuracy of human transcription was lower than the IMPROVE study which might be due to lower quality of the images. The accuracy of the SBP and DBP annotations were 93.1% and 92.6% with MAE of 4.1 and 2.7 mmHg respectively. It should be noted that, during the processing of Guatemala Set 2, we removed images for which there was no agreement among the three annotators and the images with non-readable labels. Therefore, the reported results reflect the human transcription error for readable images with at least two consistent annotations. Considering all the images, we found that 91.9% and 91.3% of the images had consistent labels by all three annotators in annotating SBP and DBP values.

In addition, we conducted a comparative analysis using a rank-sum test to assess the performance of the manual and the automatic transcriptions. In the evaluation of the Georgia IMPROVE dataset we found no statistically significant difference between the manual transcription by a single individual when compared to our automatic machine learning approach for both SBP (*p*-value = 0.9) and DBP (*p*-value = 0.9) transcriptions. Similar results were obtained for the Guatemala Set 2 dataset, with *p*-value of 0.7 and 0.4 for SBP and DBP respectively, as determined by the rank-sum test. These findings suggest that the developed automatic transcription method performs at a comparable level to manual transcription, demonstrating its potential as a reliable alternative.

## Limitations

While our study demonstrates promising results in automating the transcription of BP data from oscillometric devices in diverse settings, it is important to acknowledge certain limitations. Firstly, the performance of our model may still be influenced by varying lighting conditions, image quality, and device types beyond those tested in our study. Although we applied color correction and thresholding techniques to mitigate these issues, there are still image quality issues which cause errors in transcription. Therefore, it is crucial to address the quality assessment of images in real-time. To enhance the user experience and ensure reliable results, it is important to develop an algorithm capable of identifying image quality issues that can run on a mobile device and alert the user to retake the photo if necessary. Secondly, our study has provided valuable insights into the effectiveness of our automated image transcription technique with the specific devices used in the Guatemala and Georgia datasets. However, the diversity of BP monitoring devices available is substantial, with variations in design and display characteristics. To address this limitation and enhance the generalizability of our approach, future research should involve a more extensive evaluation on a wider selection of device types validated to be used in self-measured BP monitoring systems. This expansion would allow us to assess the adaptability and performance of our model across a diverse set of devices, taking into account potential variations in image quality, screen layouts, and digit presentation.

## Discussion and conclusion

While accuracies were generally greater than 90%, it is important to note that the error rates were generally very low, indicating that even when a transcription was incorrect, it was often in the last digit, and did not produce a clinically significant error. However, the error varied between datasets, which reflects the differences in both the lighting conditions (generally darker in less well-lit Guatemalan homes) and the different devices. In particular, without retraining, the results exhibited lower performance on a different dataset. However, device-specific training with transfer learning and device-independent digit recognition models reduced the errors down to 1-2 mmHg, demonstrating that the errors are negligible (within the error bounds of the device itself). The device-specific approach is particularly useful when the type of device being used is known and can be taken into account during the transcription process. We note that our analysis demonstrates that the mean absolute error is far below the FDA recommendation of 5 mmHg [42], which therefore makes the proposed model suitable for general use if the compound error with the chosen BP device remains within this limit. As such, we expect the continual updating of the model with more examples of a variety of BP models will eventually create a fully generalized model. In addition, we aim to enhance the model by adding an image quality assessment step which can provide real-time feedback to users to trigger recapture of data.

The integration of this technology into a clinical pathway for BP monitoring, recording and communication to healthcare professionals may enhance the management of hypertension and cardiovascular health. By automating the transcription of BP readings, this technology addresses critical challenges in capturing accurate data, particularly in low-literacy settings, and offers a range of transformative benefits. Firstly, the automated transcription reduces the potential for human errors in the recording of BP measurements. By eliminating manual data entry, the technology can help to increase quality and consistency in the data captured. This, in turn, leads to more reliable diagnostic assessments and treatment decisions. Secondly, the developed model can enhance the efficiency and convenience of BP monitoring by simplifying the process of capturing and documenting BP readings. Moreover, the automated communication of BP data to healthcare professionals enables real-time monitoring and timely intervention. In conclusion, by mitigating errors, enhancing convenience, and enabling real-time communication, this innovative solution has the potential to significantly improve patient outcomes and strengthen the communication between patients and healthcare professionals.
